# The General Medical Council

**Published:** 1902-06-07

**Authors:** 


### The General Medical Council.

The General Medical Council appears to have fallen upon somewhat evil days, for a report from its own Finance Committee shows that the funds at its disposal are no longer sufficient to meet the expenses for which they were intended to provide; that the savings of former years are melting away, and that the Council is rapidly approaching a condition of insolvency. The first Medical Act, of 1858, provided that every medical practitioner then qualified should pay a registration fee of £2, and that every practitioner registered after the passing of the Act should pay a fee of £5; the object being to furnish the Council at once with a sufficient capital and a sufficient income. From the funds hence arising, the Council was called upon to provide a staff and premises for the conduct of its business, to pay fees on a scale approved by the Treasury for the attendances and the expenses of its members, and meet certain other expenses. In addition to the freehold property in Oxford Street, there now remains an available sum of only about £13,000, while the expenses of the Council exceed its income by an amount ranging from £1,200 to £1,500 a year. Unless the income can be increased, or the expenses curtailed, the end is certainly inevitable, and can hardly be said to be remote.

In these circumstances, while there must, of course, be a general agreement that "something must be done," there is a not unnatural difference of opinion as to what the something should be. The majority of the Finance Committee declare that more money must be obtained, and they recommend the establishment of a registration fee of £1 from every medical student, and an increase of the registration fee for practitioners from £5 to £7, or to something not exceeding £10. In order to carry these recommendations into effect, a new Act of Parliament would be required, and it would have, of course, to render the registration of students compulsory, and thus, by a side wind, as the Council would manifestly be authorised to prescribe the conditions of registration, to confer upon it complete control of preliminary education. On the other hand, a minority of the committee, led by one of the most experienced members of the Council, Dr. Heron Watson, is of opinion that a remedy for the existing state of things should be first sought in a curtailment of expenditure, and that for this purpose the members should forego their fees for attendance, and that the Council should sit in the morning as well as in the afternoon, and so shorten the duration of its sessions. A comparatively new member' but one whose views would deservedly carry great weight, Dr. Norman Moore, expressed sentiments much resembling those of Cowper's Essex farmer:—

> "Quoth one, 'A rarer man than you
> In pulpit none shall hear;
> But yet, methinks, to tell you true,
> You sell it plaguey dear.'"

He actually questioned whether the medical profession and the public had sufficiently appreciated the work of the Council to think more of it worth buying at the same price; and Dr. Heron Watson believed that the suggested increase of the registration fee would be strenuously resisted by the profession. We have very little doubt that he is right, and still less that the resistance would be successful.

The simple truth is, that the Medical Council has fallen to the ground between two stools. It was established by Parliament solely for the purpose of protecting the public against incompetent or unprincipled practitioners; and yet the medical profession was made to pay for it. In order to reconcile practitioners to the impost a vague impression was created, by cloudy and irresponsible talk, that the Council would in some way become an engine working for good to the profession itself; and, when it was seen that the expectations thus created had not been and could not be fulfilled, an attempt was made to appease the resulting clamour by the concession of "direct representatives," gentlemen who increased the number of members, increased the time spent in speech-making, and consequently increased the expenses of the meetings, but who brought to the Council no increase of powers, and no additional possibility of accomplishing the various things which were expected of it by enthusiasts who had not read the Acts of Parliament from which its authority was derived. This authority was miserably inadequate even for the work which the Council was appointed to perform, the work of controlling medical education, and in most other directions was non-existent. If the Council were to go to Parliament for new powers, there cannot be a doubt that, if the question were entertained at all, the whole subject of medical education would be reconsidered, and that a central authority, if appointed, would be so constituted as to be able to defend the public against the competition of corporations. There can be no doubt, moreover, that the members of such an authority, as a matter of simple honesty, should be paid by the public for their services.

